# Early Postoperative Cell-Free DNA Reflects Renal and Hepatic Injury After Pediatric Cardiac Surgery

**DOI:** 10.3390/jcdd13060235

**Published:** 2026-05-31

**Authors:** Hiba Abuelhija, Asaf Mandel, Salmas Watad, Hai Zemmour, Eitan Keizman, David Mishaly, Esther Arfi Levy, Alain E. Serraf, Uri Pollak

**Affiliations:** 1 Section of Pediatric Critical Care, Hadassah University Medical Center, Ein Kerem, POB 12000, Jerusalem 9112001, Israel; hiba.abuelhija@gosh.nhs.uk (H.A.); asafm@hadassah.org.il (A.M.); swatad@hadassah.org.il (S.W.); 2 Faculty of Medicine, The Hebrew University of Jerusalem, Jerusalem 9112102, Israel; hai.zemmour@mail.huji.ac.il (H.Z.); estherle@hadassah.org.il (E.A.L.); selen@hadassah.org.il (A.E.S.); 3 Cardiac Intensive Care Unit, Heart and Lung Directorate, Great Ormond Street Hospital for Children NHS Foundation Trust, London WC1N 3BH, UK; 4 Department of Pediatrics, Hadassah University Medical Center, Jerusalem 9112001, Israel; 5 Pediatric and Congenital Cardiac Surgery, Edmond J. Safra International Congenital Heart Center, The Edmond and Lily Safra Children’s Hospital, Tel Hashomer, Ramat Gan 52621, Israel; ek3511@cumc.columbia.edu (E.K.); david.mishali@sheba.health.gov.il (D.M.); 6 Gray Faculty of Medical & Health Sciences, Tel Aviv University, Tel Aviv 6997801, Israel; 7 Division of Cardiac Surgery, Department of Surgery, Columbia University, New York, NY 10027, USA; 8 Pediatric and Congenital Cardiac Surgery, Hadassah University Medical Center, Jerusalem 9112001, Israel

**Keywords:** cell-free nucleic acids, acute kidney injury, liver function tests, cardiac surgical procedures, congenital heart disease

## Abstract

Circulating cell-free DNA (cfDNA) is released during tissue injury and systemic inflammation, but its association with postoperative organ injury following pediatric cardiac surgery remains incompletely defined. We evaluated the relationship between early postoperative cfDNA levels and acute kidney injury (AKI) and biochemical hepatic injury in children undergoing open-heart surgery with cardiopulmonary bypass (CPB). This retrospective observational cohort study included 50 pediatric patients (<18 years) who underwent CPB at a tertiary congenital heart center between 2017 and 2018. Plasma cfDNA concentrations were measured perioperatively, with the 6 h postoperative value analyzed as an early biomarker window. AKI was classified using Kidney Disease: Improving Global Outcomes criteria, and hepatic injury was assessed using serial liver enzyme measurements. cfDNA levels increased significantly within 6 h after CPB and were higher in patients with more severe AKI. Six-hour cfDNA concentrations correlated with postoperative creatinine, urea, alanine aminotransferase, and aspartate aminotransferase. In multivariable regression analyses adjusting for cardiopulmonary bypass duration, aortic cross-clamp time, and preoperative oxygen saturation, cfDNA at 6 h remained independently associated with AKI severity and peak liver enzyme levels. These exploratory findings suggest that early postoperative cfDNA elevation is associated with AKI severity and biochemical hepatic injury after pediatric cardiac surgery with CPB. Larger prospective studies are needed to determine its independent predictive value and clinical utility.

## 1. Introduction

Circulating cell-free DNA (cfDNA) has emerged as a promising biomarker in diverse clinical contexts, including oncology, transplantation, prenatal medicine, and critical illness, due to its capacity to provide real-time information about tissue injury and systemic inflammation [1,2,3,4]. In healthy individuals, plasma cfDNA levels are low and tightly regulated, but increase rapidly following acute tissue damage, necrosis, apoptosis, or inflammatory activation [2,4]. This dynamic release and clearance of cfDNA has made it a candidate for early detection and monitoring of pathological processes in critically ill patients [3,5].

Pediatric open-heart surgery with cardiopulmonary bypass (CPB) imposes substantial physiological and immunological stress, often resulting in a profound inflammatory response and increased risk of multi-organ dysfunction [6,7,8,9]. Acute kidney injury (AKI) is observed in up to 30–50% of pediatric patients following CPB, and is associated with increased morbidity, length of intensive care, and mortality [6,10,11,12]. Hepatic injury, frequently manifested as elevations in aminotransferases and cholestatic enzymes, is also common after pediatric cardiac surgery and may occur in parallel with renal dysfunction [7,13]. The mechanisms underlying these complications are multifactorial, involving hemodynamic instability, ischemia–reperfusion injury, and, notably, the robust inflammatory milieu elicited by CPB [7,9,13].

CPB triggers innate and adaptive immune activation with release of cytokines, chemokines, and damage-associated molecular patterns (DAMPs) [7,8,14]; among these, cfDNA functions both as a cellular-injury marker and as a DAMP that engages Toll-like receptor 9 (TLR9) and the cyclic GMP–AMP synthase-stimulator of interferon genes (cGAS-STING) pathway to amplify systemic inflammation [1,14,15,16,17], a dual role that underpins its potential for early detection and risk stratification of post-surgical complications [2,15,18].

Despite its promise, the clinical utility of cfDNA measurement for early identification of AKI and hepatic injury after pediatric CPB remains incompletely defined [3,5]. Although prior studies have linked postoperative cfDNA elevations to adverse outcomes in adult and pediatric cardiac surgery [18,19,20], prospective data detailing the timing, magnitude, and prognostic implications of cfDNA relative to specific organ injury in children are scarce. To address this gap, we evaluated early postoperative cfDNA kinetics and their association with AKI and hepatic injury in children undergoing open-heart surgery with CPB, hypothesizing that early cfDNA elevations would associate with injury severity and illuminate the interplay between inflammation, cfDNA release, and multi-organ dysfunction.

## 2. Patients and Methods

### 2.1. Study Design and Setting

This observational retrospective cohort study analyzed data from a cfDNA biobank that was prospectively established at the Edmond J. Safra International Congenital Heart Center at Safra Children’s Hospital in Tel Hashomer, Israel, between August 2017 and August 2018. The study was conducted in accordance with the Declaration of Helsinki and approved by the Institutional Review Board (IRB) at Sheba Medical Center (#4993-18-SMC). Written informed consent was obtained from parents or legal guardians prior to any study procedures.

### 2.2. Study Population

Inclusion criteria for the cfDNA biobank consisted of pediatric patients (age < 18 years) undergoing cardiac surgery with CPB. Patients were excluded if they had pre-existing conditions that could independently affect cfDNA concentrations, such as systemic infections or autoimmune diseases. The final cohort included 50 patients meeting these criteria.

### 2.3. Data Collection

Clinical, demographic, and laboratory perioperative data were extracted from the institutional electronic medical records (see Supplementary Table S1).

Cell-free DNA Measurement. Peripheral blood samples for cfDNA analysis were obtained at predefined perioperative time points: preoperatively (within 24 h prior to surgery), and postoperatively at 0, 6, 12, and 24 h after completion of CPB. Sampling time points were selected as part of the original cfDNA biobank protocol to characterize perioperative cfDNA kinetics: preoperative sampling provided an individual baseline; time 0 captured immediate release at the end of CPB; and 6, 12, and 24 h samples assessed early postoperative kinetics, persistence, and clearance during the first postoperative day. cfDNA extraction, analysis of methylation and quantification were previously described [21] and expressed as copies per milliliter (copies/mL). Although cfDNA was measured at all predefined perioperative time points in the biobank, the present analysis focused on early values at the end of CPB/time 0 and 6 h postoperatively, as these were most relevant to evaluating the association between early cfDNA release and subsequent renal and hepatic injury while avoiding temporal ambiguity from later measurements.

### 2.4. Definition of Outcomes

#### 2.4.1. Renal Injury

AKI was staged using the Kidney Disease: Improving Global Outcomes (KDIGO) classification, based on changes in serum creatinine and urine output in the first 72 h postoperatively. Additional renal parameters included perioperative urea, uric acid, and fluid balance at 12 and 24 h.

#### 2.4.2. Hepatic Injury

Hepatic injury was assessed via serial measurements of ALT, AST, ALP, LDH, and their respective ratios. Maximal postoperative values were recorded.

#### 2.4.3. Other Clinical Outcomes

Other secondary outcomes included maximal postoperative lactate, minimal bicarbonate, and composite major morbidity (ECMO, in-hospital mortality).

#### 2.4.4. Sample Size Justification

This study was a secondary analysis of a prospectively established cfDNA biobank; therefore, the sample size was determined by the available biobank cohort rather than by an a priori power calculation for the present analysis. With n = 50, the study had approximately 80% power to detect a correlation of about r = 0.39 at a two-sided alpha of 0.05. To reduce the risk of overfitting, multivariable models were restricted to a small number of prespecified clinically relevant predictors. The findings should therefore be interpreted as exploratory and hypothesis-generating.

### 2.5. Statistical Analysis

Descriptive statistics were reported as mean ± standard deviation (SD), median [interquartile range, IQR], or number (percentage), as appropriate. Group comparisons were performed using the Kruskal–Wallis test for nonparametric data and chi-squared or Fisher’s exact test for categorical variables. Correlations between cfDNA and laboratory parameters were assessed using Spearman’s rank correlation coefficient, with adjustment for potential confounders (CPB time, aortic cross-clamp time) via partial correlation. The association analyses focused on cfDNA at time 0 and 6 h because these early postoperative measurements best preceded renal and hepatic injury, while broader longitudinal cfDNA kinetics from the same biobank cohort were reported previously [21].

Multivariable regression analyses were conducted to identify independent predictors of AKI (KDIGO stage, ordinal) and hepatic injury (maximal ALT and AST, linear), including cfDNA (log-transformed), CPB and cross-clamp times, and preoperative oxygen saturation as candidate variables. Model fit was assessed by the coefficient of determination (R^2^). All statistical analyses were performed using Python’s (v3.10) scientific computing libraries. A two-sided *p*-value < 0.05 was considered statistically significant.

## 3. Results

### 3.1. Patient Characteristics

A total of 50 pediatric patients who underwent cardiac surgery with CPB were analyzed (Supplementary Table S1). The median operative weight was 4550 g [IQR: 3155–7575], and the median preoperative oxygen saturation was 85% [IQR: 76.5–90]. Surgical complexity was STS-EACTS median 3 [IQR 2–4], range 1–5. Maximal vasoactive-inotropic score median was 15.0 [IQR 5.6–25.0], mechanical ventilation time median was 23.5 h [IQR 7.25–96.0], ventilation-free days at 28 days median was 26.5 [IQR 24–27], hospital length of stay median was 8 days [IQR 6–16], ECMO in 2/50 patients, and in-hospital mortality occurred in 4/50 patients (Supplementary Table S2). Demographic and baseline laboratory data are summarized in Table 1.

Summary of demographic, preoperative, operative, and selected laboratory variables for the study cohort (N = 50). Data are presented as mean (SD), median [IQR], or range as appropriate.

### 3.2. Renal Injury Outcomes

Acute kidney injury, as classified by KDIGO stage, was present in most patients: 38% stage 1, 28% stage 2, and 4% stage 3. Postoperative maximal creatinine and urea levels increased with KDIGO stage, with mean postoperative creatinine of 0.59 ± 0.26 mg/dL and urea of 56.1 ± 22.1 mg/dL.

**Figure 1:**
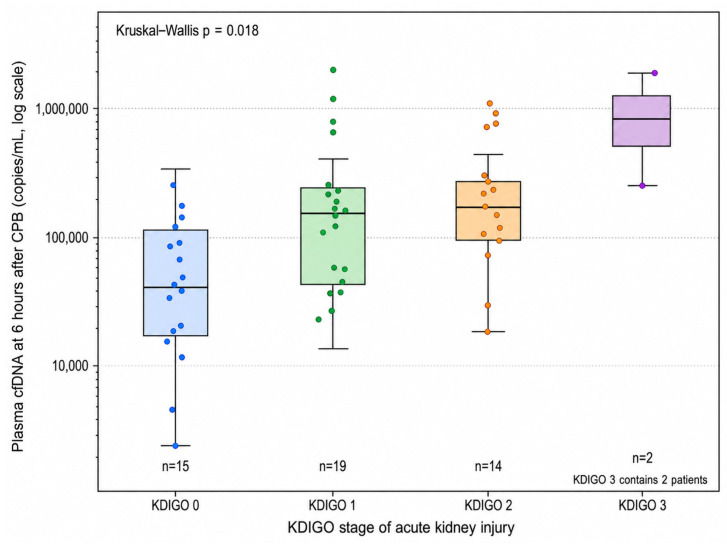
Plasma cfDNA at 6 Hours after CPB by KDIGO stage of acute kidney injury. Legend: Boxplot illustrating cfDNA concentrations measured 6 h after CPB, stratified by KDIGO stage. Circles denote statistical outliers. A significant trend toward higher cfDNA levels with increasing KDIGO stage was observed (Kruskal–Wallis *p* = 0.018). cfDNA is presented on a logarithmic scale due to skewed distribution.

cfDNA levels rose significantly with increasing AKI severity (Figure 1). Kruskal–Wallis analysis demonstrated significant group differences (*p* = 0.018 for cfDNA at 6 h, *p* = 0.008 at 12 h).

Partial Spearman correlations (adjusted for cardiopulmonary bypass and cross-clamp time) confirmed moderate associations between cfDNA (6 h) and postoperative creatinine (ρ = 0.32, *p* = 0.026), urea (ρ = 0.33, *p* = 0.019), and fluid balance at 24 h (ρ = 0.28, *p* = 0.049) (Table 2).

Values represent exploratory partial Spearman correlations adjusted for CPB duration and aortic cross-clamp duration. Correlation coefficients indicate weak-to-modest associations and should not be interpreted as evidence of causality or stand-alone predictive performance.

### 3.3. Hepatic Injury Outcomes

**Figure 2:**
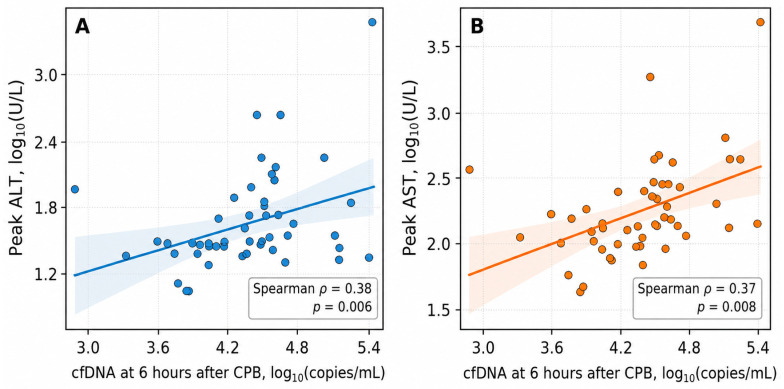
Association Between log_10_(cfDNA at 6 Hours Post-CPB) and log_10_(ALT, AST). Legend: Scatterplots showing exploratory associations between log10-transformed cfDNA measured 6 h after CPB and log10-transformed peak postoperative ALT (**A**) and AST (**B**). Each point represents one patient. Both ALT and AST are positively associated with cfDNA (ALT: ρ = 0.38, *p* = 0.006; AST: ρ = 0.37, *p* = 0.008), suggesting that higher early cfDNA levels may reflect more severe hepatic injury. The observed associations were modest and should be interpreted as exploratory rather than evidence of causality or stand-alone predictive utility.

Liver injury was frequent, with marked postoperative rises in ALT, AST, LDH, and ALP (Table 2). cfDNA at 6 h was significantly correlated with postoperative ALT (ρ = 0.38, *p* = 0.006), AST (ρ = 0.37, *p* = 0.008), LDH (ρ = 0.28, *p* = 0.050), and ALP (ρ = 0.29, *p* = 0.044) (Figure 2).

### 3.4. Regression Analyses and Key Predictors

AKI (KDIGO as outcome):

Multivariable ordinal logistic regression demonstrated that higher log10-transformed cfDNA at 6 h was independently associated with greater AKI severity by KDIGO stage (OR 4.89, 95% CI 1.25–19.04, *p* = 0.022), after adjustment for CPB duration, aortic cross-clamp duration, and preoperative oxygen saturation (Table 3). Sensitivity analysis excluding ECMO-treated patients yielded directionally consistent results, although precision was limited by the small cohort size and the small number of patients with KDIGO stage 3 and this ordinal regression analysis was considered exploratory.

KDIGO stage was analyzed as an ordered categorical outcome using ordinal logistic regression. Odds ratios greater than 1 indicate higher odds of being in a more severe KDIGO category. The ordinal model included cardiopulmonary bypass duration, aortic cross-clamp duration, log10-transformed cfDNA at 6 h postoperatively, and preoperative oxygen saturation. Hepatic injury outcomes were analyzed using multivariable linear regression models with log10-transformed postoperative maximal ALT and AST as dependent variables, adjusted for the same covariates. For hepatic injury models, only the coefficient for log10 (cfDNA at 6 h) is shown, as this was the primary predictor of interest. cfDNA, cell-free DNA; CPB, cardiopulmonary bypass; KDIGO, Kidney Disease: Improving Global Outcomes; ALT, alanine aminotransferase; AST, aspartate aminotransferase; OR, odds ratio; CI, confidence interval.

Hepatic Injury (ALT/AST as outcomes):

Regression models showed that cfDNA at 6 h (log-transformed) was the only significant independent predictor of postoperative ALT and AST (Table 3), with CPB, X-clamp, and preoperative O2 saturation not significant.

## 4. Discussion

This study represents a secondary analysis of data from the prospectively established cfDNA biobank and addresses the clinical question regarding early postoperative total cfDNA kinetics and non-cardiac organ injury, specifically KDIGO-defined AKI and biochemical hepatic injury after pediatric CPB. It demonstrates that early postoperative cfDNA elevations are associated with AKI severity and biochemical hepatic injury in children undergoing cardiac surgery with CPB. These findings support cfDNA as a candidate early biomarker of postoperative tissue-injury burden. Because this was an observational study, the data do not establish whether cfDNA contributes mechanistically to post-CPB organ dysfunction although they are consistent with prior mechanistic hypotheses. Our results are broadly consistent with, but extend, the prior literature: few studies have integrated comprehensive laboratory phenotyping with serial cfDNA measurements in pediatric populations, and cardiovascular reports demonstrating cfDNA patterns for myocardial injury [22] and general tissue injury [23] underscore the novelty of our simultaneous evaluation of renal and hepatic outcomes.

Our findings should also be interpreted in the context of recent pediatric cardiac surgery studies evaluating cfDNA as a marker of postoperative risk. Tanem et al. reported that nuclear cfDNA after pediatric cardiothoracic surgery was associated with adverse postoperative events, supporting the concept that cfDNA reflects clinically meaningful postoperative injury burden in this population [20]. Similarly, Scott et al. found that elevated nuclear and mitochondrial cfDNA measurements were associated with mortality after infant cardiac surgery [24]. The present study extends these observations by focusing specifically on renal and biochemical hepatic injury after CPB and by evaluating early perioperative cfDNA measurements in relation to KDIGO-defined AKI and postoperative liver enzyme abnormalities. Thus, while prior studies linked cfDNA to global adverse outcomes and mortality, our data suggest that early postoperative cfDNA may also reflect specific non-cardiac organ injury patterns after pediatric CPB.

Our data indicate that postoperative cfDNA concentrations increase stepwise with AKI severity (as per KDIGO stage) and modestly correlate with both maximal postoperative creatinine and urea levels, as well as with indices of hepatic injury (ALT, AST, LDH, ALP). Notably, the rise in cfDNA precedes the peak of conventional laboratory markers, supporting its potential as an early warning biomarker for organ dysfunction. This temporal advantage could enable earlier identification of high-risk patients and facilitate timely therapeutic intervention. While earlier investigations in critically ill adults and children have linked plasma cfDNA to adverse outcomes such as AKI, hepatic dysfunction, sepsis, and mortality [20,24,25,26,27], our study uniquely applies this approach to pediatric cardiac surgery, extending prognostic utility beyond cardiac-specific injury patterns to multi-organ dysfunction. While encouraging, the correlation analyses should be interpreted cautiously. Although 6 h cfDNA was associated with renal and hepatic laboratory indices, the correlation coefficients were modest. These findings suggest that cfDNA may reflect postoperative tissue-injury burden but is unlikely to function as a stand-alone biomarker. Its potential utility may lie in integration with clinical, operative, and laboratory variables in broader risk-stratification models.

Beyond its diagnostic utility, previously published data showed that cfDNA may also contribute mechanistically to post-CPB organ injury. Although not studied directly in our current research, CPB is known to trigger a complex systemic inflammatory response, driven by blood contact with artificial surfaces, ischemia/reperfusion injury, and innate immune activation, a syndrome sometimes termed “post-pump” or “systemic inflammatory response syndrome (SIRS).” cfDNA, released during necrosis, apoptosis, and NETosis, is increasingly recognized as a damage-associated molecular pattern (DAMP) that can amplify this inflammatory cascade [28]. Experimental studies demonstrate that cfDNA can activate Toll-like receptor 9 (TLR9) and the cyclic GMP-AMP synthase-stimulator of interferon genes (cGAS-STING) pathway, leading to robust production of type I interferons and pro-inflammatory cytokines [29,30]. In our study, the association of early postoperative cfDNA elevations with both AKI and biochemical hepatic injury, after adjustment for CPB duration, cross-clamp time, and preoperative oxygen saturation, raises the possibility that cfDNA reflects broader postoperative inflammatory and tissue-injury burden. This observation is consistent with prior mechanistic hypotheses but does not establish causal involvement of cfDNA in organ dysfunction.

The identification of cfDNA as a sensitive early biomarker of organ injury holds promise for risk stratification, therapeutic targeting, and personalized medicine. Early postoperative cfDNA measurements could identify children at high risk for AKI and hepatic injury, enabling vigilant monitoring and proactive management; this utility has been demonstrated in sepsis where cfDNA levels correlate with disease severity [20]. If cfDNA is mechanistically involved in injury, interventions to promote its clearance (e.g., DNase infusion) or inhibit downstream signaling (e.g., TLR9 or STING antagonists) may offer novel therapies, as suggested by early-phase studies in sepsis and trauma. Moreover, integrating cfDNA kinetics with clinical and molecular data could inform precision medicine strategies, tailoring interventions to individual patient risk; advances in cfDNA fragmentomics and artificial intelligence-driven analyses further enhance opportunities for personalized risk assessment [31,32].

### 4.1. cfDNA: Marker, Mediator, or Both?

An ongoing debate is whether cfDNA merely reflects the extent of tissue injury or actively drives inflammation and organ dysfunction [33]. Our interval analysis revealed that the most robust associations between cfDNA and hepatic injury occurred in the early perioperative phase (preop to time 0 interval), while changes from time 0 to 6 h were less predictive. This temporal pattern is compatible with a hypothesis in which surgical injury, reperfusion, and postoperative inflammation contribute to cfDNA release and organ-injury biomarkers. However, the present study was not designed to test a mechanistic ‘two-hit’ model, and such mechanisms require dedicated experimental or translational validation.

Recent evidence suggests both theories have merit, with cfDNA function being highly context dependent [34]. In physiological conditions, cfDNA likely functions primarily as cellular waste, requiring efficient clearance through the reticuloendothelial system, with 70–90% processed by liver Kupffer cells within hours [27]. However, when clearance mechanisms are overwhelmed or when released in excessive quantities during pathological states such as cardiac surgery, the same molecules can function as inflammatory DAMPs [28].

Despite these findings, important questions remain regarding the mechanisms by which cfDNA induces organ-specific injury, whether through direct tubular toxicity, endothelial activation, or immune cell recruitment, and its interactions with other damage-associated molecular patterns, cytokines, and genetic susceptibility in post-cardiopulmonary bypass injury; prospective validation in larger, multicenter cohorts is required to confirm our results and to define optimal cfDNA thresholds for clinical use. Moreover, emerging technologies such as fragmentomics to determine cfDNA tissue origins [31] and machine learning algorithms that integrate cfDNA levels, fragmentation patterns, and clinical variables [32] may enhance predictive accuracy, while interventional trials targeting cfDNA clearance or downstream signaling are needed to establish whether the modulation of cfDNA pathways improves patient outcomes.

### 4.2. Limitations

This study has several limitations. First, it is a retrospective, single-center secondary analysis of a prospectively established cfDNA biobank with a limited sample size. The study was not powered for definitive predictive modeling, and the regression analyses should be interpreted as exploratory. Second, although the model was intentionally restricted to a small number of prespecified clinically relevant variables, the cohort included only 50 patients—and only two patients with KDIGO stage 3 AKI. This sparse severe-event count may lead to unstable estimates and wide confidence intervals, and the findings should therefore be considered exploratory and hypothesis-generating rather than definitive evidence of independent predictive performance. Third, ECMO may affect creatinine kinetics, fluid balance, inflammatory activation, and KDIGO classification and therefore remains a potential source of residual confounding. Fourth, although cfDNA was associated with postoperative renal and hepatic injury, the observed correlations with laboratory indices were modest and should be considered exploratory. Finally, although mixed-effects modeling allowed use of the serial cfDNA measurements, the findings require validation in larger prospective multicenter cohorts.

## 5. Conclusions

Early postoperative cfDNA elevation was associated with AKI severity and biochemical hepatic injury after pediatric cardiac surgery with CPB. These findings support cfDNA as a candidate early biomarker of postoperative tissue-injury burden. Larger prospective studies are needed to determine its independent predictive value, clinical thresholds, and potential mechanistic role.

## Figures and Tables

**Table 1:** Descriptive statistics for key demographic, clinical, and laboratory variables.

Variable	Mean	SD	Median	IQR	Range
Operation Weight (g)	5891	4285	4550	3155–7575	2300–25,000
Preop O2 saturation (%)	83.6	11.6	85.0	76.5–90.0	40–100
Preop Creatinine (mg/dL)	0.34	0.17	0.3	0.24–0.40	0.15–1.14
Postop max Creatinine	0.59	0.26	0.56	0.40–0.66	0.24–1.72
Preop Urea (mg/dL)	23.7	12.4	23.0	15.3–29.0	5.0–60.0
Postop max Urea	56.1	22.1	51.0	44.5–63.8	19.0–113.0
Preop ALT (U/L)	24.0	34.1	17.0	13.0–21.0	6.0–238.0
Postop max ALT	121.3	413.5	31.0	24.5–68.0	11.0–2924.0
Preop AST (U/L)	44.4	31.2	39.0	30.3–45.8	15.0–208.0
Postop max AST	322.9	729.8	142.5	106.5–266.0	43.0–5028.0
Preop Total cfDNA	8070	12,465	5068	3309–9126	755–86,961
Postop cfDNA (6 h)	43,923	57,435	26,641	10,942–40,740	758–260,580

**Table 2:** Exploratory partial Spearman correlations between 6 h cfDNA and renal/hepatic laboratory indices (adjusted for CPB and cross-clamp time).

Comparison	Spearman ρ	*p*-Value
cfDNA 6 h vs. Postop max Creatinine	0.32	0.026
cfDNA 6 h vs. Postop max Urea	0.33	0.019
cfDNA 6 h vs. Postop max ALT	0.38	0.006
cfDNA 6 h vs. Postop max AST	0.37	0.008
cfDNA 6 h vs. Postop max LDH	0.28	0.05
cfDNA 6 h vs. Postop max ALP	0.29	0.044
cfDNA 6 h vs. Fluid Balance 24 h	0.28	0.049

**Table 3:** Predictors of clinical outcomes following pediatric cardiac surgery.

Outcome	Model	Predictor	Estimate	SE	Test Statistic	*p*-Value	95% CI
AKI severity (KDIGO stage)	Ordinal logistic regression	CPB duration, min	OR 1.04	--	z = 2.30	0.022	1.01–1.07
		Aortic cross-clamp duration, min	OR 0.96	--	z = −2.34	0.019	0.93–0.99
		log10(cfDNA at 6 h)	OR 4.89	--	z = 2.29	0.022	1.25–19.04
		Preoperative O2 saturation, %	OR 1.03	--	z = 0.88	0.379	0.97–1.09
Hepatic injury—ALT	Multivariable linear regression	log10(cfDNA at 6 h)	β 0.33	0.14	t = 2.30	0.026	0.04–0.62
Hepatic injury—AST	Multivariable linear regression	log10(cfDNA at 6 h)	β 0.35	0.11	t = 3.08	0.004	0.12–0.58

## Data Availability

The datasets used and/or analyzed during the current study are available as Supplementary Table S1.

## Supplementary Materials

The following supporting information can be downloaded at: https://www.mdpi.com/article/10.3390/jcdd13060235/s1, Table S1: The datasets used and/or analyzed during the current study; Table S2: Summary of the death cases within the study cohort.

## Abbreviations

The following abbreviations are used in this manuscript:

AKI	Acute Kidney Injury
ALT	Alanine Aminotransferase
AST	Aspartate Aminotransferase
ALP	Alkaline Phosphatase
cfDNA	Cell-Free DNA
CPB	Cardiopulmonary Bypass
DAMP	Damage-Associated Molecular Pattern
ECMO	Extracorporeal Membrane Oxygenation
ICU	Intensive Care Unit
KDIGO	Kidney Disease: Improving Global Outcomes
LDH	Lactate Dehydrogenase
NETosis	Neutrophil Extracellular Trap Formation
PICU	Pediatric Intensive Care Unit
RACHS-1	Risk Adjustment for Congenital Heart Surgery
STS-EACTS	Society of Thoracic Surgeons-European Association for Cardio-Thoracic Surgery
TLR9	Toll-Like Receptor 9
cGAS-STING	Cyclic GMP-AMP Synthase-Stimulator of Interferon Genes

## References

[1] Jahr S., Hentze H., Englisch S., Hardt D., Fackelmayer F.O., Hesch R.D., Knippers R. (2001). DNA fragments in the blood plasma of cancer patients: Quantitations and evidence for their origin from apoptotic and necrotic cells. Cancer Res..

[2] Lo Y.M., Chiu R.W. (2011). Plasma nucleic acid analysis by massively parallel sequencing: Pathological insights and diagnostic implications. J. Pathol..

[3] Saukkonen K., Lakkisto P., Pettilä V., Varpula M., Karlsson S., Ruokonen E., Pulkki K., for the Finnsepsis Study Group (2008). Cell-free plasma DNA as a predictor of outcome in severe sepsis and septic shock. Clin. Chem..

[4] Schwarzenbach H., Hoon D.S., Pantel K. (2011). Cell-free nucleic acids as biomarkers in cancer patients. Nat. Rev. Cancer.

[5] Rainer T.H., Wong L.K., Lam W., Yuen E., Lam N.Y., Metreweli C., Lo Y.D. (2003). Prognostic use of circulating plasma nucleic acid concentrations in patients with acute stroke. Clin. Chem..

[6] Krawczeski C.D., Goldstein S.L., Woo J.G., Wang Y., Piyaphanee N., Ma Q., Bennett M., Devarajan P. (2011). Temporal relationship and predictive value of urinary acute kidney injury biomarkers after pediatric cardiopulmonary bypass. J. Am. Coll. Cardiol..

[7] Chew M.S., Brandslund I., Brix-Christensen V., Ravn H.B., Hjortdal V.E., Pedersen J., Hjortholm K., Hansen O.K., Tønnesen E. (2001). Tissue injury and the inflammatory response to pediatric cardiac surgery with cardiopulmonary bypass: A descriptive study. Anesthesiology.

[8] Stocker C.F., Shekerdemian L.S., Visvanathan K., Skinner N., Brizard C.P., Carlin J.B., Horton S.B., Penny D.J. (2004). Cardiopulmonary bypass elicits a prominent innate immune response in children with congenital heart disease. J. Thorac. Cardiovasc. Surg..

[9] Bierer J., Stanzel R., Henderson M., Sett S., Sapp J., Andreou P., Marshall J.S., Horne D. (2023). Novel inflammatory mediator profile observed during pediatric heart surgery with cardiopulmonary bypass and continuous ultrafiltration. J. Transl. Med..

[10] Blinder J.J., Goldstein S.L., Lee V.V., Baycroft A., Fraser C.D., Nelson D., Jefferies J.L. (2012). Congenital heart surgery in infants: Effects of acute kidney injury on outcomes. J. Thorac. Cardiovasc. Surg..

[11] Li S., Krawczeski C.D., Zappitelli M., Devarajan P., Thiessen-Philbrook H., Coca S.G., Kim R.W., Parikh C.R. (2011). Incidence, risk factors, and outcomes of acute kidney injury after pediatric cardiac surgery: A prospective multicenter study. Crit. Care Med..

[12] Bouchard J., Macedo E., Soroko S., Chertow G.M., Himmelfarb J., Ikizler T.A., Paganini E.P., Mehta R.L., Program to Improve Care in Acute Renal Disease (2010). Comparison of methods for estimating glomerular filtration rate in critically ill patients with acute kidney injury. Nephrol. Dial. Transplant..

[13] Kehl T., Biermann D., Briem-Richter A., Schoen G., Olfe J., Sachweh J.S., Fischer L., Schaefer H., Kozlik-Feldmann R., Gottschalk U. (2021). Impact of hepatopathy in pediatric patients after surgery for complex congenital heart disease. PLoS ONE.

[14] Itagaki K., Kaczmarek E., Lee Y.T., Tang I.T., Isal B., Adibnia Y., Sandler N., Grimm M.J., Segal B.H., Otterbein L.E. (2015). Mitochondrial DNA Released by Trauma Induces Neutrophil Extracellular Traps. PLoS ONE.

[15] Rainer T.H. (2001). Plasma DNA, prediction and post-traumatic complications. Clin. Chim. Acta.

[16] Zhang Q., Raoof M., Chen Y., Sumi Y., Sursal T., Junger W., Brohi K., Itagaki K., Hauser C.J. (2010). Circulating mitochondrial DAMPs cause inflammatory responses to injury. Nature.

[17] Oka T., Hikoso S., Yamaguchi O., Taneike M., Takeda T., Tamai T., Oyabu J., Murakawa T., Nakayama H., Nishida K. (2012). Mitochondrial DNA that escapes from autophagy causes inflammation and heart failure. Nature.

[18] Qi Y., Uchida T., Yamamoto M., Yamamoto Y., Kido K., Ito H., Ohno N., Asahara M., Yamada Y., Yamaguchi O. (2016). Perioperative Elevation in Cell-Free DNA Levels in Patients Undergoing Cardiac Surgery: Possible Contribution of Neutrophil Extracellular Traps to Perioperative Renal Dysfunction. Anesthesiol. Res. Pract..

[19] Nakahira K., Kyung S.Y., Rogers A.J., Gazourian L., Youn S., Massaro A.F., Quintana C., Osorio J.C., Wang Z., Zhao Y. (2013). Circulating mitochondrial DNA in patients in the ICU as a marker of mortality: Derivation and validation. PLoS Med..

[20] Tanem J.M., Scott J.P., Hoffman G.M., Niebler R.A., Tomita-Mitchell A., Stamm K.D., Liang H.-L., North P.E., Bertrandt R.A., Woods R.K. (2023). Nuclear Cell-Free DNA Predicts Adverse Events After Pediatric Cardiothoracic Surgery. Ann. Thorac. Surg..

[21] Pollak U., Zemmour H., Shaked E., Magenheim J., Fridlich O., Korach A., Serraf A.E., Mishaly D., Glaser B., Shemer R. (2023). Novel cfDNA Methylation Biomarkers Reveal Delayed Cardiac Cell Death after Open-heart Surgery. J. Cardiovasc. Transl. Res..

[22] Zemmour H., Planer D., Magenheim J., Moss J., Neiman D., Gilon D., Korach A., Glaser B., Shemer R., Landesberg G. (2018). Non-invasive detection of human cardiomyocyte death using methylation patterns of circulating DNA. Nat. Commun..

[23] Hummel E.M., Hessas E., Müller S., Beiter T., Fisch M., Eibl A., Wolf O.T., Giebel B., Platen P., Kumsta R. (2018). Cell-free DNA release under psychosocial and physical stress conditions. Transl. Psychiatry.

[24] Scott J.P., Tanem J.M., Tomita-Mitchell A., Hoffman G.M., Niebler R.A., Liang H.L., Simpson P.M., Stamm K.D., North P.E., Mitchell M.E. (2022). Elevated nuclear and mitochondrial cell-free deoxyribonucleic acid measurements are associated with death after infant cardiac surgery. J. Thorac. Cardiovasc. Surg..

[25] Charoensappakit A., Sae-Khow K., Rattanaliam P., Vutthikraivit N., Pecheenbuvan M., Udomkarnjananun S., Leelahavanichkul A. (2023). Cell-free DNA as diagnostic and prognostic biomarkers for adult sepsis: A systematic review and meta-analysis. Sci. Rep..

[26] Duvvuri B., Lood C. (2019). Cell-Free DNA as a Biomarker in Autoimmune Rheumatic Diseases. Front Immunol..

[27] Kustanovich A., Schwartz R., Peretz T., Grinshpun A. (2019). Life and death of circulating cell-free DNA. Cancer Biol. Ther..

[28] Korabecna M., Zinkova A., Brynychova I., Chylikova B., Prikryl P., Sedova L., Neuzil P., Seda O. (2020). Cell-free DNA in plasma as an essential immune system regulator. Sci. Rep..

[29] Wan D., Jiang W., Hao J. (2020). Research Advances in How the cGAS-STING Pathway Controls the Cellular Inflammatory Response. Front Immunol..

[30] Saber M.M., Monir N., Awad A.S., Elsherbiny M.E., Zaki H.F. (2022). TLR9: A friend or a foe. Life Sci..

[31] Qi T., Pan M., Shi H., Wang L., Bai Y., Ge Q. (2023). Cell-Free DNA Fragmentomics: The Novel Promising Biomarker. Int. J. Mol. Sci..

[32] Moser T., Kühberger S., Lazzeri I., Vlachos G., Heitzer E. (2023). Bridging biological cfDNA features and machine learning approaches. Trends Genet..

[33] Heitzer E., Auinger L., Speicher M.R. (2020). Cell-Free DNA and Apoptosis: How Dead Cells Inform About the Living. Trends Mol. Med..

[34] Han D.S.C., Lo Y.M.D. (2021). The Nexus of cfDNA and Nuclease Biology. Trends Genet..

