# Supplementary material for: Early Postoperative Cell-Free DNA Reflects Renal and Hepatic Injury After Pediatric Cardiac Surgery
Source: J Cardiovasc Dev Dis. 2026 May 31;13(6):235. doi: 10.3390/jcdd13060235 (PMC13300183; doi:10.3390/jcdd13060235)
Supplement: Supplementary file 1 [file jcdd-13-00235-s001.zip › cfDNA_JCDD_supp Table S1.pdf]

Supplementary Table S1. Patient-level clinical, operative, biomarker, and outcome data

This table summarizes the 50 pediatric cardiac surgery patients included in the cohort. Patient identifiers are study row numbers only; dates of birth and operation dates were intentionally omitted.

Supplementary Table S1A. Baseline demographic and preoperative characteristics

Patient	Sex	Diagnosis	Arch anomaly	Preop MV	Preop CPR	Preop VIS	PGE1	Preop O2Sat	BAS	Preop LVFS	Op age (d)	Op wt (g)
1	Male	TGA + VSD	No	No	No	0.0	No	80	No	35	4	3140
2	Male	Transposition of the Great Arteries	No	No	No	0.0	Yes	78	Yes	35	4	2700
3	Male	Transposition of the Great Arteries	No	No	No	0.0	Yes	88	No	35	5	3700
4	Male	Transposition of the Great Arteries	No	Yes	No	10.0	Yes	85	No	35	11	2700
5	Female	TGA + VSD	No	Yes	No	30.0	Yes	85	Yes	35	8	3100
6	Male	Transposition of the Great Arteries	No	No	No	0.0	Yes	85	No	35	9	3500
7	Female	Single ventricle with CoA	Yes	Yes	Yes	7.5	Yes	88	Yes	35	25	4500
8	Male	L-TGA	No	No	No	0.0	No	78	No	35	245	11800
9	Male	Truncus arteriosus	No	No	No	0.0	No	100	No	45	2002	16000
10	Male	TGA + VSD	No	No	No	0.0	Yes	85	No	35	7	4100
11	Male	TOF	No	No	No	0.0	No	95	No	40	405	7600
12	Female	TOF	No	No	No	0.0	No	100	No	35	4085	25000
13	Male	TOF	No	No	No	0.0	No	84	No	35	665	9500
14	Male	Transposition of the Great Arteries	No	Yes	No	0.0	No	80	Yes	30	139	6100
15	Male	Unbalanced AVC with TAPVR	No	No	No	0.0	No	74	No	35	531	10000
16	Female	TAPVR	No	No	No	0.0	No	85	No	35	7	3100
17	Female	VSD	No	No	No	0.0	No	100	No	43	323	7150
18	Male	HLHS	No	Yes	No	0.0	Yes	85	No	35	26	2900
19	Male	HLHS	No	Yes	No	5.5	Yes	80	No	35	30	3300

Patient	Sex	Diagnosis	Arch anomaly	Preop MV	Preop CPR	Preop VIS	PGE1	Preop O2Sat	BAS	Preop LVFS	Op age (d)	Op wt (g)
20	Female	TGA + VSD	No	No	No	0.0	No	90	No	35	706	9100
21	Female	HLHS	No	No	No	0.0	Yes	90	No	35	9	2300
22	Female	HLHS	No	No	No	0.0	Yes	95	No	35	26	2300
23	Male	AS/AI	No	No	No	0.0	No	100	No	43	383	8500
24	Male	TOF	No	No	No	0.0	No	100	No	35	217	7500
25	Male	HRV	Yes	No	No	0.0	No	76	No	35	338	8600
26	Female	HLHS	No	No	No	0.0	No	85	No	35	2261	17000
27	Female	HLHS	No	No	No	0.0	No	72	No	35	258	6300
28	Male	HLHS	No	No	No	0.0	Yes	88	No	35	6	2650
29	Male	VSD	No	No	No	0.0	No	98	No	35	78	5000
30	Male	TOF	No	No	No	0.0	No	75	No	35	355	5500
31	Male	Transposition of the Great Arteries	No	No	No	0.0	Yes	70	No	35	6	2700
32	Male	HRV	Yes	No	No	0.0	Yes	75	No	35	3	3900
33	Male	TOF	No	No	No	0.0	Yes	80	No	35	35	4000
34	Female	VSD	No	No	No	0.0	No	100	No	33	249	5000
35	Female	HRV	Yes	No	No	0.0	No	75	No	35	239	4900
36	Female	Unbalanced AVC with TAPVR	No	Yes	No	0.0	Yes	75	No	35	18	2600
37	Male	HLHS	No	Yes	No	10.0	Yes	75	No	35	16	3200
38	Male	TOF	No	No	No	0.0	No	55	No	45	252	7700
39	Male	HRV	Yes	No	No	0.0	No	80	No	35	331	6200
40	Female	HRV	Yes	Yes	No	0.0	No	40	No	35	95	3300
41	Female	Transposition of the Great Arteries	No	No	No	0.0	Yes	75	No	35	13	2800
42	Female	HRV	Yes	No	No	0.0	No	80	No	35	188	6150
43	Male	HLHS	No	No	No	0.0	Yes	90	No	35	4	3300

Patient	Sex	Diagnosis	Arch anomaly	Preop MV	Preop CPR	Preop VIS	PGE1	Preop O2Sat	BAS	Preop LVFS	Op age (d)	Op wt (g)
44	Male	HRV	Yes	No	No	0.0	No	96	No	35	229	8200
45	Female	VSD	Yes	Yes	No	2.0	Yes	93	No	35	27	3300
46	Male	TOF	No	No	No	0.0	No	95	No	47	58	4650
47	Female	TOF	No	No	No	0.0	Yes	80	No	35	96	4600
48	Male	Transposition of the Great Arteries	No	Yes	No	0.0	Yes	85	No	35	41	2500
49	Female	Unbalanced AVC with TAPVR	No	Yes	No	0.0	Yes	85	No	35	26	3300
50	Female	HLHS	No	No	No	0.0	No	70	No	35	198	7600

Supplementary Table S1B. Operative variables and risk categories

Patient	Redo	Surgical procedure	Arch repair	CPB (min)	XCLMP (min)	Min temp	RACHS-1	STS-EACTS	ABC score
1	No	ASO + VSD repair	No	148	130	28.0	4	4	11.0
2	No	Arterial Switch Operation	No	149	125	34.0	3	3	10.0
3	No	Arterial Switch Operation	No	98	86	28.0	3	3	10.0
4	No	Arterial Switch Operation	No	55	35	35.0	3	3	10.0
5	No	ASO + VSD repair	No	75	42	36.0	4	4	11.0
6	No	Arterial Switch Operation	No	56	39	36.0	3	3	10.0
7	No	CoA repair + PAB	Yes	0	0	36.0	3	4	6.0
8	Yes	Double Switch	No	188	153	29.0	5	5	13.8
9	Yes	RVPAC + AVR	No	123	86	35.0	4	3	7.5
10	No	ASO + VSD repair	No	72	50	36.0	4	4	11.0
11	No	TOF repair	No	57	44	36.0	2	2	8.0
12	Yes	RVPAC	No	44	0	36.0	3	3	7.5
13	No	TOF repair	No	41	29	36.0	2	2	8.0
14	Yes	Retraining + ASO	No	60	42	36.0	4	4	11.0
15	Yes	1 & 1/2 ventricles (with Glenn)	No	148	133	28.0	2	2	9.0
16	No	TAPVR repair	No	56	45	36.0	4	4	9.0
17	No	VSD repair	No	28	20	36.0	2	1	6.0
18	No	CoA repair + PAB	No	95	51	36.0	3	4	6.0
19	Yes	Norwood	No	70	38	36.0	6	5	14.5
20	No	ASO + VSD repair	No	223	169	28.0	4	4	11.0
21	No	Norwood	No	114	88	28.0	6	5	14.5
22	No	Norwood	No	78	44	36.0	6	5	14.5
23	Yes	Ross/Konno	No	148	142	28.0	4	4	12.5

Patient	Redo	Surgical procedure	Arch repair	CPB (min)	XCLMP (min)	Min temp	RACHS-1	STS-EACTS	ABC score
24	No	TOF repair	No	46	36	36.0	2	2	8.0
25	Yes	Glenn	Yes	26	0	36.0	2	2	6.8
26	Yes	Fontan	No	37	0	35.0	3	2	9.0
27	Yes	Glenn	No	84	0	36.0	2	2	6.8
28	No	Norwood	No	78	40	36.0	6	5	14.5
29	No	VSD repair	No	42	30	36.0	2	1	6.0
30	Yes	TOF repair	No	79	58	36.0	2	2	8.0
31	No	Arterial Switch Operation	No	104	89	28.0	3	3	10.0
32	No	BTT shunt	Yes	20	0	36.0	3	4	6.3
33	No	TOF repair	No	59	48	34.0	2	2	8.0
34	No	VSD repair	No	42	26	36.0	2	1	6.0
35	Yes	Glenn	Yes	48	0	36.0	2	2	6.8
36	No	BTT shunt	No	94	25	30.0	3	4	6.3
37	No	Norwood	No	78	43	36.0	6	5	14.5
38	No	TOF repair	No	72	58	36.0	2	2	8.0
39	Yes	Glenn	Yes	67	41	36.0	2	2	6.8
40	No	BTT shunt	Yes	31	0	35.0	3	4	6.3
41	No	Arterial Switch Operation	No	53	36	36.0	3	3	10.0
42	Yes	Glenn	Yes	37	0	36.0	2	2	6.8
43	No	Norwood	No	71	39	36.0	6	5	14.5
44	Yes	Glenn	Yes	33	0	36.0	2	2	6.8
45	No	VSD repair	Yes	84	48	35.0	2	1	6.0
46	No	TOF repair	No	57	49	34.0	2	2	8.0
47	No	BTT shunt	No	45	0	36.0	3	4	6.3
48	No	Arterial Switch Operation	No	109	92	30.0	3	3	10.0

Patient	Redo	Surgical procedure	Arch repair	CPB (min)	XCLMP (min)	Min temp	RACHS-1	STS-EACTS	ABC score
49	No	BTT shunt	No	77	35	31.0	3	4	6.3
50	Yes	Glenn	No	97	0	32.0	2	2	6.8

Supplementary Table S1C. cfDNA measurements

Patient	Preop total cfDNA	Preop cardiac cfDNA	T0 total cfDNA	T0 cardiac cfDNA	T6 total cfDNA	T6 cardiac cfDNA	T12 total cfDNA	T12 cardiac cfDNA	T24 total cfDNA	T24 cardiac cfDNA
1	8320	107	86961	12317	138932	19217	28084	1951	13275	414
2	6920	78	43054	4946	37996	5887	9902	803	13982	306
3	10974	44	65039	4685	57994	6261	12684	999	11247	424
4	4078	45	18920	2688	23080	3005	19218	1899	10020	405
5	12446	24	137302	13442	129381	8630	133305	6636	213120	14634
6	6558	51	49813	8371	38723	8064	12053	1605	5333	319
7	8840	159	24179	514	6981	195	7580	304		
8	1854	1	29088	2798	174164	3021			24907	3090
9	1582	6	55873	4072	36360	3205	14154	1092	15214	731
10	12735	118	76419	12429	29378	4544	10917	1095	6964	320
11	6199	3	31349	1803	32663	1250	14331	238		
12	2462	13	22498	269	24341	165	7878	98	8891	124
13	1805	4	55057	5099	25204	2144	175740	2733	53104	2176
14	13472	107	60391	7272	107656	8044	90011	3189	32499	623
15	3414	13	32724	2776	30704	3178	22361	3089	17762	2221
16	5068	13	52250	7460	14684	1921	11353	714	7151	142
17	4973	20	33143	3022	31346	1815	12085	315	3054	67
18	3860	165	75568	20301	30361	8196	18863	4970		
19	16362	154	48722	2525	28078	1457	17107	770	11514	245
20	4072	127	86355	6557	44931	3924	19756	1440	55267	1941
21			60539	9591	243158	20081	220180	22048	59630	5167
22	13817	26	102414	15784	43026	8919	38178	10048	33734	4326
23	2618	6	39875	4429	33881	5321	42875	5413	39239	3014

Patient	Preop total cfDNA	Preop cardiac cfDNA	T0 total cfDNA	T0 cardiac cfDNA	T6 total cfDNA	T6 cardiac cfDNA	T12 total cfDNA	T12 cardiac cfDNA	T24 total cfDNA	T24 cardiac cfDNA
24	3345	5	107155	10503	10908	883	13762	776	8802	328
25	3309	2738	9040	7818	13158	6130	14804	10975		
26	2716		2396	730	260580	64297	211549	21450	228260	74063
27	5345	4478	43026	23454	49328	19697	27270	20499		
28	5649	3672	83477	16866	142300	37777	201633	12490	239370	35083
29	9568	3058	37976	9398	11044	8332	10257	8468	26543	4194
30	3412	2141	58934	36795	14924	13086	10209	1241	24673	21691
31	10411	9172	5355	3005	2091	958	1818	1626	2140	1549
32	755	624	8772	6001	7350	4639	6597	5165	4935	470
33	5545	13	11196	2218	3909	781	6408	761	4136	427
34	1345	37	32724	5068	8740	836	6532	270	3337	81
35	2121	17	2586	106	21271	1099	13256	667	21059	330
36	5818	150	14393	1990	24584	3094	28309	1972	46056	1830
37	10408	41	22018	1762	39996	3190	23965	1665	15684	1046
38	9126	37	13835	2837	5909	752	6472	494	7763	402
39	4681	205	21968	1164	22180	640	17726	659	13797	330
40	86961	1560	113019	3187	758	16	122412	1679	96203	2282
41	1803	9	18879	3313	10817	1859	5136	532	4363	378
42	16782	163	10847	668	17938	1133	14196	555	14580	370
43	23634	513	49450	3050	32724	2206	57772	3510	46541	2361
44	5000	1	20453	873	12650	256	9217	182	5954	44
45	9072	70	53126	11825	51662	10482	19998	3444	12226	1205
46	1288	8	18483	3620	7878	1271	4893	317	2666	439
47	1927	35	13890	853	5499	375	9799	394	11648	195

Patient	Preop total cfDNA	Preop cardiac cfDNA	T0 total cfDNA	T0 cardiac cfDNA	T6 total cfDNA	T6 cardiac cfDNA	T12 total cfDNA	T12 cardiac cfDNA	T24 total cfDNA	T24 cardiac cfDNA
48	5352	14	21117	3752	4766	112	7631	864	10757	748
49	3545	8	9772	1170	40988	1328	15784	554	12090	336
50	4100	143	15243	428	9151	279	24283	570		

Supplementary Table S1D. Postoperative biomarkers and outcomes

Patient	Max lactate	Min HCO3	Max troponin	Max AST	Max ALT	Max LDH	Max ALP	ALT/ALP	Max Cr	Max urea	Uric acid	Cr ratio	KDIGO	Max VIS	FB 12h	FB 24h	POD1 LVFS	VFD-28	MV time (h)	Discharge LVFS	LOS (d)	ECMO	Exitus
1	52.0	15.6	14.5	133	21	761	196	0.11	0.72	62	6.0	1.60	1	23.0	-8	-69	25	26	47	36	6	No	No
2	63.0	19.4	10.6	159	127	1605	161	0.79	0.92	51	7.0	1.51	1	12.5	68	-17	35	27	20	37	7	No	No
3	75.0	20.0		115	45	785	244	0.18	0.57	31	4.0	1.21	0	15.0	75	118	24	27	21	42	7	No	No
4	52.0	21.4	9.4	96	24	987	153	0.16	0.57	52	5.6	2.11	2	20.0	397	423	35	26	26	41	7	No	No
5	158.0	12.6	47.1	648	35	2513	107	0.33	1.72	93	10.5	2.77	2	55.0	50	303	17	0	96	0	4	No	Yes
6	47.0	22.3	10.0	91	26	589	419	0.06	0.58	19	5.4	1.66	1	12.5	47	99	35	26	25	36	5	No	No
7	33.0	18.5	1.6	43	11	691	76	0.14	0.37	44	2.9	1.12	0	17.5	-118	101	35	19	210	35	18	No	No
8	55.0	17.3	78.0	443	69	1942	130	0.53	0.60	63	12.0	2.73	2	35.1	392	374	35	21	168	35	15	No	No
9	33.0	20.5	73.5	284	34	991	161	0.21	0.57	49	7.4	1.97	1	12.6	-384	-488	25	27	3	30	6	No	No
10	21.0	20.1	15.5	231	16	960	298	0.05	0.51	56	7.8	1.59	1	15.0	79	177	20	25	53	35	10	No	No
11	29.0	19.2	7.8	221	65	673	247	0.26	0.41	53	10.0	1.21	0	10.0	378	363	35	27	17	38	5	No	No
12	77.0	20.6	3.2	111	53	455	324	0.16	0.53	50	5.8	1.32	0	5.0	186	403	36	27	6	45	13	No	No
13	82.0	11.4	53.0	254	96	818	161	0.60	0.77	102	10.9	2.85	2	10.0	321	235	35	27	4	36	8	No	No
14	85.0	15.6	10.8	202	180	798	373	0.48	0.77	83	11.3	1.71	1	25.0	121	267	35	24	96	38	8	No	No
15	56.0	16.8	78.0	440	179	1316	397	0.45	0.48	113	15.2	1.92	1	37.5	29	135	35	24	96	35	21	No	No
16	39.0	24.7	2.1	99	28	670	157	0.18	0.49	57	6.2	1.26	0	2.5	82	22	34	27	9	33	5	No	No
17	23.0	22.0	7.7	140	31	555	345	0.09	0.43	40	8.0	0.69	0	0.0	32	-207	33	27	7	37	4	No	No
18	81.0	24.7	66.1	293	29	962	112	0.26	0.69	50	4.8	2.65	2	25.0	119	227	35	24	96	35	4	No	No
19	75.0	26.6	12.4	1848	432	2797	178	2.43	0.69	50	5.8	1.73	1	35.0	30	-13	35	0	768	0	32	No	Yes
20	43.0	17.4	68.0	418	433	1424	220	1.97	0.60	104	10.3	2.40	2	25.0	264	608	25	0	114	0	30	No	Yes
21	173.0	16.1	16.4	142	22	714	300	0.07	0.82	51	4.2	1.55	1	30.0	-14	9	35	23	101	35	16	No	No
22	75.0	17.7	15.2	153	54	646	263	0.21	0.47	48	5.3	1.27	0	50.0	44	91	35	17	244	35	24	No	No
23	44.0	20.1	78.0	472	53	1184	204	0.26	0.53	62	9.6	1.89	1	20.0	178	244	30	27	19	39	7	No	No
24	26.0	20.9	21.6	143	30	572	168	0.18	0.32	29	7.9	1.52	1	10.0	5	197	35	27	8	34	4	No	No
25	33.0	19.8	2.7	75	50	811	134	0.37	0.32	53	6.6	1.33	0	0.0	-410	-571	35	27	4	35	6	No	No

Patient	Max lactate	Min HCO3	Max troponin	Max AST	Max ALT	Max LDH	Max ALP	ALT/ALP	Max Cr	Max urea	Uric acid	Cr ratio	KDIGO	Max VIS	FB 12h	FB 24h	POD1 LVFS	VFD-28	MV time (h)	Discharge LVFS	LOS (d)	ECMO	Exitus
26	155.0	15.8	4.5	5028	2924	5540	177	16.52	1.13	109	12.1	3.65	3	30.0	1084	1236	35	27	5	35	27	No	No
27	27.0	21.1	10.9	137	20	708	270	0.07	0.48	35	7.3	1.60	1	5.0	209	46	35	27	1	35	4	No	No
28	172.0	7.8		449	27	1183	104	0.26	0.98	70	9.0	2.39	2	105.0	179	382	25	0	48	0	2	Yes	Yes
29	30.0	19.8	11.2	132	28	616	396	0.07	0.33	60	6.8	1.74	1	10.0	167	305	23	27	22	38	6	No	No
30	28.0	19.1	52.0	248	31	1369	219	0.14	0.36	41	7.2	1.89	1	17.0	65	114	34	27	20	35	19	No	No
31	79.0	19.2	17.0	112	23	741	193	0.12	0.66	42	5.2	1.38	0	19.0	17	9	33	25	50	40	7	No	No
32	23.0	21.2	0.5	47	11	424	163	0.07	0.66	48	5.5	1.16	0	2.5	19	98	35	26	28	35	5	No	No
33	19.0	20.8	13.0	167	31	714	755	0.04	0.40	47	5.4	1.67	1	5.0	47	-3	35	27	17	35	6	No	No
34	18.0	22.6	9.8	123	24	609	142	0.17	0.30	46	6.2	1.50	1	5.0	36	138	31	27	4	38	8	No	No
35	34.0	18.8	5.6	95	23	633	261	0.09	0.24	23	4.8	1.26	0	5.0	-62	63	35	27	4	35	7	No	No
36	83.0	16.9	15.0	68	31	934	129	0.24	0.65	88	5.2	1.97	1	10.0	120	210	35	23	116	35	18	No	No
37	162.0	8.2	12.1	191	111	998	495	0.22	0.37	49	6.6	2.06	2	25.0	41	56	35	20	173	35	18	No	No
38	36.0	15.0	20.6	154	13	974	329	0.04	0.31	26	11.2	1.03	0	0.0	-58	-181	37	27	7	46	7	No	No
39	27.0	21.0	4.4	136	41	859	236	0.17	0.54	71	13.7	2.08	2	30.0	-145	-114	35	27	3	28	10	No	No
40	176.0	16.3	13.2	366	92	1702	171	0.54	1.14	69	8.8	1.00	0	25.0	147	-97	20	22	132	35	24	Yes	No
41	73.0	16.2	7.0	90	19	630	189	0.10	0.65	28	4.7	1.44	0	15.0	82	61	35	27	22	45	7	No	No
42	30.0	18.7	3.0	128	77	1274	221	0.35	0.64	67	14.3	2.78	2	0.0	119	137	35	27	8	35	8	No	No
43	121.0	12.7	7.6	136	71	853	170	0.42	0.79	64	7.3	2.93	2	25.0	176	299	35	26	46	35	14	No	No
44	63.0	17.6	4.6	78	28	763	261	0.11	0.46	59	9.1	1.77	1	10.0	-202	-143	30	27	3	35	16	No	No
45	74.0	21.0	4.5	270	35	1540	244	0.14	0.60	48	6.0	3.33	3	15.0	93	178	32	26	48	35	43	No	No
46	31.0	21.0	7.8	183	30	654	416	0.07	0.32	50	4.9	1.52	1	15.0	134	179	35	25	51	44	8	No	No
47	127.0	15.3	1.7	58	24	561	359	0.07	0.41	26	8.4	2.73	2	7.5	168	345	35	25	71	35	14	No	No
48	56.0	19.0	13.5	102	30	702	179	0.17	0.39	35	2.1	1.26	0	10.0	-160	-215	30	27	22	35	9	No	No
49	178.0	16.1	12.2	288	148	1424	311	0.48	0.36	57	9.0	2.12	2	2.5	13	-127	35	7	504	35	70	No	No
50	30.0	19.8	2.9	105	29	879	226	0.13	0.63	82	11.7	2.25	2	5.0	47	56	30	27	5	30	9	No	No

Notes and abbreviations

ABC score, Aristotle Basic Complexity Score; ALP, alkaline phosphatase; ALT, alanine aminotransferase; AS/AI, aortic stenosis/aortic insufficiency; ASO, arterial switch operation; AST, aspartate aminotransferase; BAS, balloon atrial septostomy; BTT, Blalock-Taussig-Thomas; CoA, coarctation of the aorta; CPB, cardiopulmonary bypass; CPR, cardiopulmonary resuscitation; Cr, creatinine; ECMO, extracorporeal membrane oxygenation; HLHS, hypoplastic left heart syndrome; HCO3, bicarbonate; HRV, hypoplastic right ventricle; KDIGO, Kidney Disease: Improving Global Outcomes; LDH, lactate dehydrogenase; LOS, length of hospital stay; LVFS, left ventricular fractional shortening; MV, mechanical ventilation; PAB, pulmonary artery banding; PGE1, prostaglandin E1; POD, postoperative day; PO, postoperative; RACHS-1, Risk Adjustment for Congenital Heart Surgery; RVPAC, right ventricle-to-pulmonary artery conduit; STS-EACTS, Society of Thoracic Surgeons-European Association for Cardio-Thoracic Surgery; TAPVR, total anomalous pulmonary venous return; TGA, transposition of the great arteries; TOF, tetralogy of Fallot; VFD-28, ventilator-free days at 28 days; VIS, vasoactive-inotropic score; VSD, ventricular septal defect; XCLMP, aortic cross-clamp duration.

cfDNA values are presented in copies/ml. Categorical risk variables (RACHS-1, STS-EACTS, KDIGO) are shown as categories rather than continuous variables.

Preoperative LVFS categories in the field key: normal 26-45%; mild 20-25%; moderate 15-19%; severe <15%. The dataset contained numeric LVFS values; numeric values are shown.
