# Supplementary material for: Early Postoperative Cell-Free DNA Reflects Renal and Hepatic Injury After Pediatric Cardiac Surgery
Source: J Cardiovasc Dev Dis. 2026 May 31;13(6):235. doi: 10.3390/jcdd13060235 (PMC13300183; doi:10.3390/jcdd13060235)
Supplement: Supplementary file 1 [file jcdd-13-00235-s001.zip › cfDNA_JCDD_supp Table S2.pdf]

Supplementary Table S2. Deaths Cases

This table summarizes the 4 pediatric cardiac surgery patients included in the cohort who died.

Diagnosis	Procedure	Age at surgery	Weight	CPB duration	X-clamp duration	KDIGO stage	ECMO
TGA + VSD	ASO + VSD repair	8 days	3.1 kg	75 min	42 min	2	No
HLHS	Norwood	30 days	3.3 kg	70 min	38 min	1	No
TGA + VSD	ASO + VSD repair	706 days	9.1 kg	223 min	169 min	2	No
HLHS	Norwood	6 days	2.65 kg	78 min	40 min	2	Yes
